# A field realistic model to assess the effects of pesticides residues and adulterants on honey bee gene expression

**DOI:** 10.1371/journal.pone.0302183

**Published:** 2024-06-26

**Authors:** Noëmie El Agrebi, Lina De Smet, Caroline Douny, Marie-Louise Scippo, Lidija Svečnjak, Dirk C. de Graaf, Claude Saegerman

**Affiliations:** 1 Research Unit of Epidemiology and Risk analysis applied to Veterinary sciences (UREAR-ULiège), Fundamental and Applied Research for Animal and Health (FARAH) Center, Faculty of Veterinary Medicine, University of Liège, Liège, Belgium; 2 Faculty of Sciences, Laboratory of Molecular Entomology and Bee Pathology, Ghent University (UGent), Ghent, Belgium; 3 Faculty of Sciences, Honeybee Valley, Ghent University (UGent), Ghent, Belgium; 4 Laboratory of Food Analysis, Department of Food Sciences, Faculty of Veterinary Medicine, Fundamental and Applied Research for Animals & Health (FARAH) Center, Faculty of Veterinary Medicine, University of Liège, Liège, Belgium; 5 University of Zagreb, Faculty of Agriculture, Department of Fisheries, Apiculture, Wildlife Management and Special Zoology, Zagreb, Croatia; Gomal University, PAKISTAN

## Abstract

While studies on the sublethal effects of chemical residues in beeswax on adult honey bees are increasing, the study protocols assessing the impacts on honey bee brood in realistic conditions still need to be investigated. Moreover, little is known about the residue’s effect on gene expression in honey bee brood. This study reports the effects of chlorpyriphos-ethyl, acrinathrin and stearin worker pupae exposure through contaminated or adulterated beeswax on the gene expression of some key health indicators, using a novel *in vivo* realistic model. Larvae were reared in acrinathrin (12.5, 25, 10 and 100 ppb) and chlorpyriphos-ethyl (5, 10, 500 and 5000 ppb) contaminated or stearin adulterated beeswax (3, 4, 5, 6 and 9%) in newly formed colonies to reduce the influence of external factors. On day 11, mortality rates were assessed. Honey bee pupae were extracted from the comb after 19 days of rearing and were analysed for the gene expression profile of four genes involved in the immune response to pathogens and environmental stress factors (*Imd*, *dorsal*, *domeless* and *defensin*), and two genes involved in detoxifications mechanisms (CYP6AS14 and CYP9Q3). We found no linear relation between the increase in the pesticide concentrations and the brood mortality rates, unlike stearin where an increase in stearin percentage led to an exponential increase in brood mortality. The immune system of pupae raised in acrinathrin contaminated wax was triggered and the expression of CYP6AS14 was significantly upregulated (exposure to 12.5 and 25 ppb). Almost all expression levels of the tested immune and detoxification genes were down-regulated when pupae were exposed to chlorpyrifos-contaminated wax. The exposure to stearin triggered the immune system and detoxification system of the pupae. The identification of substance-specific response factors might ultimately serve to identify molecules that are safer for bees and the ecosystem’s health.

## Introduction

In modern beekeeping, removable frames are used to allow beekeepers to extract honey and inspect the hive without damaging the comb. The comb can then be relocated inside the hive and reused. This reduces the time and energy that honey bees spend on producing wax. In good beekeeping practice, brood combs are ideally replaced after three years [1]. To produce new foundations, the old comb wax together with wax cell cappings are recycled by melting.

Honey bees may carry pesticide residues back to the hive, particularly from plant protection products, as they bring pollen, nectar, water, honeydew, or propolis collected from their environment (e.g. [2–8]). For many years, the use of pesticides was considered the main pest management strategy. Within the hive, both types of residues can end up in beeswax of the existing and recycled combs [9–11], it has also been shown that pesticide degradation products or metabolites can have higher toxicity than their parent compound [12]. When applied in the environment, in the hive or present in the wax foundation, apart from their immediate lethal effects, pesticides can generate insidious sublethal effects that impact the behaviour [13–15], the reproduction, the development of colonies, and can generate resistance of the organisms chronically exposed to their active substances [16].

Another emerging problem of beeswax for honey bees is its adulteration by the addition of natural or synthetic substances of wide availability and low price. The most common sources of adulterations are hydrocarbons from paraffin and microcrystalline waxes, triglycerides from palmitic acid, fat and hardened beef tallow, industrially produced fatty acids (palmitic, stearic acid), long-chain alcohols (C16-C18), and C32-C36 synthetic esters [17–20].

The contamination and adulteration of beeswax is an issue that has been reported lately on several occasions in the scientific literature [2, 21–31]. The effects of these contaminated or adulterated beeswax foundations seem to be the main cause of poor brood and colony development [32, 33]. Contaminants in beeswax affect highly sensitive honey bee larvae and pupae through two pathways, orally; as contaminants can migrate from beeswax to royal jelly and by direct contact as larvae and pupae develop in wax cells [2].

Among pesticide residues, the organophosphate insecticide chlorpyrifos, also known as chlorpyrifos-ethyl (contact acute LD_50_ [worst case from 24, 48 and 72-hour values] = 0.068 μg/bee) is one of the most commonly detected agrochemicals in beeswax [34]. It was found in the Belgian apiaries with a prevalence of 5.9% in 2021 and 2022, and 13.5% in 2016 [26]. The high prevalence of chlorpyrifos has been confirmed in recent years by other studies [3, 8, 35, 36]. Organophosphate insecticides, like chlorpyrifos and coumaphos, act on the insect nervous system by inhibiting acetylcholinesterase, the enzyme responsible for deactivating the neurotransmitter acetylcholine in insect central nervous system synapses [37]. Several effects such as an increase in apoptosis have been reported in larvae treated with chlorpyrifos orally compared to untreated larvae [38]. Contact exposure to field-relevant concentrations of chlorpyrifos in combination with chlorothalonil resulted in decreased sperm viability in sexually mature males [39]. Although little is known about the effects of chlorpyrifos on honey bees and their pupae, it was shown to have significant synergetic effects when combined with other pesticides, resulting in high larval mortality [40, 41].

Another pesticide residue group, with a high environmental impact, to which honey bees are often exposed is pyrethroids. One of them is acrinathrin (contact acute LD_50_ [worst case from 24, 48 and 72-hour values] = 0.084 μg/bee) It was used in the past to control *Varroa*, but is no longer authorized due to its high toxicity and the emergence of resistance. Acrinathrin was found in larvae after direct contact with contaminated beeswax [42]. The standard Hazard Quotient (HQ) value of acrinathrin which expresses its potential toxicity to bees, exceeds the trigger value of 50, indicating the need for further refinement of the risk assessment of this substance [43]. Acrinathrin application in field and semi-field tests increased honey bee mortality immediately after and up to 3 days after application. Nevertheless, no notable effects were observed on honey bee colony strength or bee brood development. To reduce honey bee exposure immediately after the application of acrinathrin and for up to 4 days thereafter, risk reduction measures are recommended [43].

Recently, stearin, a mixture of stearic and palmitic acids, was reported as one of the main adulterants of beeswax [44]. Moreover, preliminary studies conducted in Belgium [33], Poland [32] and Germany [30] confirmed an association between the presence of stearin and detrimental effects on bee brood.

Previous research on the effects of pesticide residues by contact exposure on honey bee health has typically focused on adult honeybees, in *in vitro* conditions. However, field experiments are essential to study the risk assessment of pesticide impact on immature bees and brood development. This study focuses on the response of pupae reared in contaminated beeswax with field-realistic concentrations of chlorpyrifos-ethyl, acrinathrin and stearin. The expression profile of some key immune and detoxification genes was followed. The three major immune response pathways were studied by following the expression level of the genes *relish* (involved in the Imd pathway), *domeless* (involved in the Janus kinase-signal transducer and activator of transcription [Jak-STAT] pathway) and *dorsal* (involved in the Toll pathway) [45]. *Defensin* was used as a marker for the production of antimicrobial peptides. Chemicals may trigger some detoxification pathways with CYP6AS14 and CYP9Q3 as key enzymes in the degradation process. Oxidative stress generated by exposure to chemicals can be mapped by following the expression of catalase and glutathione-S-transferase (GST) coding genes. The results of expression profiling will provide insight into how the pupae respond to and deal with exposure to chemicals.

## Materials and methods

### Virgin beeswax selection

Virgin beeswax was purchased from an organic beeswax producer and analysed using a multi-residue analysis by gas chromatography-tandem mass spectrometry (GC-MS/MS) and liquid chromatography-tandem mass spectrometry (LC-MS/MS) methods encompassing 294 distinct compounds with detection limits (LOD) of 0.003 mg/kg and limits of quantification (LOQ) that are of 0.01 mg/kg for the majority of the compounds. The analysis was carried out in an independent laboratory in Germany (Intertek Food Services GmbH) according to the European EN 15662 method (CEN 2008), using a common analytical protocol (QuEChERS) designed for the analysis of food materials and suitably adapted [26]. The virgin beeswax was also analysed by Fourier transform infrared spectroscopy (FTIR) coupled with a single-reflection diamond Attenuated Total Reflectance (ATR) system (FTIR-ATR spectroscopy) according to the methods for adulteration detection developed by Svečnjak et al. (2019) [20]. Both analyses confirmed the absence of any chemical contamination by pesticides or adulteration in the foundation wax.

### Beeswax contamination and residue analysis

**Fig 1:**
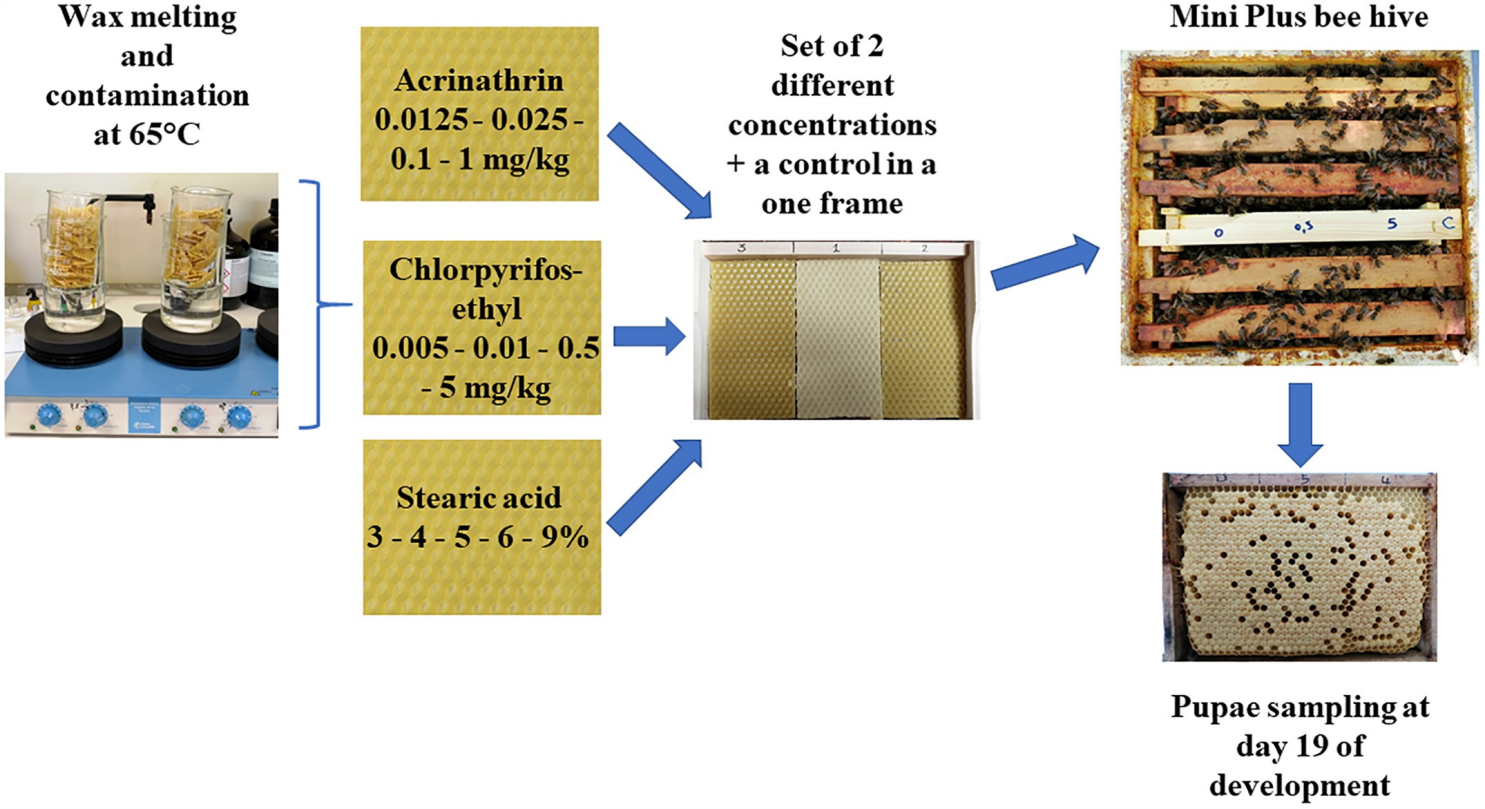
Experimental set to obtain 18 emerging honey bees (3 emerging bees per concentration or percentage x 6 replicates) in contaminated and adulterated beeswax with four concentrations of acrinathrin and chlorpyrifos-ethyl and stearin adulterated beeswax.

To obtain beeswax foundations with a chlorpyrifos-ethyl concentration of 5 and 10 ppb in the first year (2020) and of 500 and 5000 ppb in the second year (2021), 9.2 mg of the substance (chlorpyrifos-ethyl, purity 99.49%, purchased from LGC) was added to 9.2 ml of dimethyl sulfoxide (DMSO) (99.9%), and then it was diluted 10 times. A certain volume of the diluted solution (25, 50, 2500 or 25 000 μl) was added to 500 g of the melted beeswax and homogenised using a magnetic stirring bar on a heating plate at 65°C for 5 minutes, to obtain the concentration of 5, 10, 500 and 5000 ppb, respectively. The same procedure was applied to obtain beeswax foundations with an acrinathrin concentration of 12.5 and 25 ppb in the first year and 10 and 100 ppb in the second year. The new beeswax foundations were formed with a foundation mould (Fig 1). The concentrations used are similar to the respective concentrations of chlorpyrifos-ethyl [26, 46, 47] and acrinathrin [46–48] found in commercial or beekeepers beeswax.

### Beeswax adulteration

Stearin (Radiacid 0464 –CAS No. 67701-03-5) was obtained from Oleon, NV. The stearin used is a well-balanced mixture of stearic and palmitic acids. The ratio between the two compounds determines the melting point. In our case, the melting point was 55 °C. The stearin was composed of 58% of palmitic acid (C16) and 40% of stearic acid (C18). Stearin was added to the melted beeswax at the following percentages (w/w) the first year: 3, 6 and 9%, and 3, 4 and 5% the second year. The effective percentages of stearin in the beeswax after supplementation were evaluated by FTIR-ATR spectroscopy.

### Study site and colony establishment

We conducted our study on the site of the Faculty of Veterinary Medicine of the University of Liège, Belgium (50°34’30.913", 5°35’43.832"). The Western honey bee (*Apis mellifera* L.) subspecies that was used was the dark European honey bee (*Apis mellifera mellifera*). A naked swarm was first treated with oxalic acid to eliminate phoretic *Varroa* mites. The swarms were headed by naturally mated sister queens. The naked swarm was introduced into a Mini Plus hive [49] on virgin beeswax frames. Once colonies were well established and queens were laying eggs, one experimental frame per colony supporting a control comb section (virgin beeswax) and two contaminated comb sections were placed in the middle of the nest.

### Experimental frame

**Fig 2:**
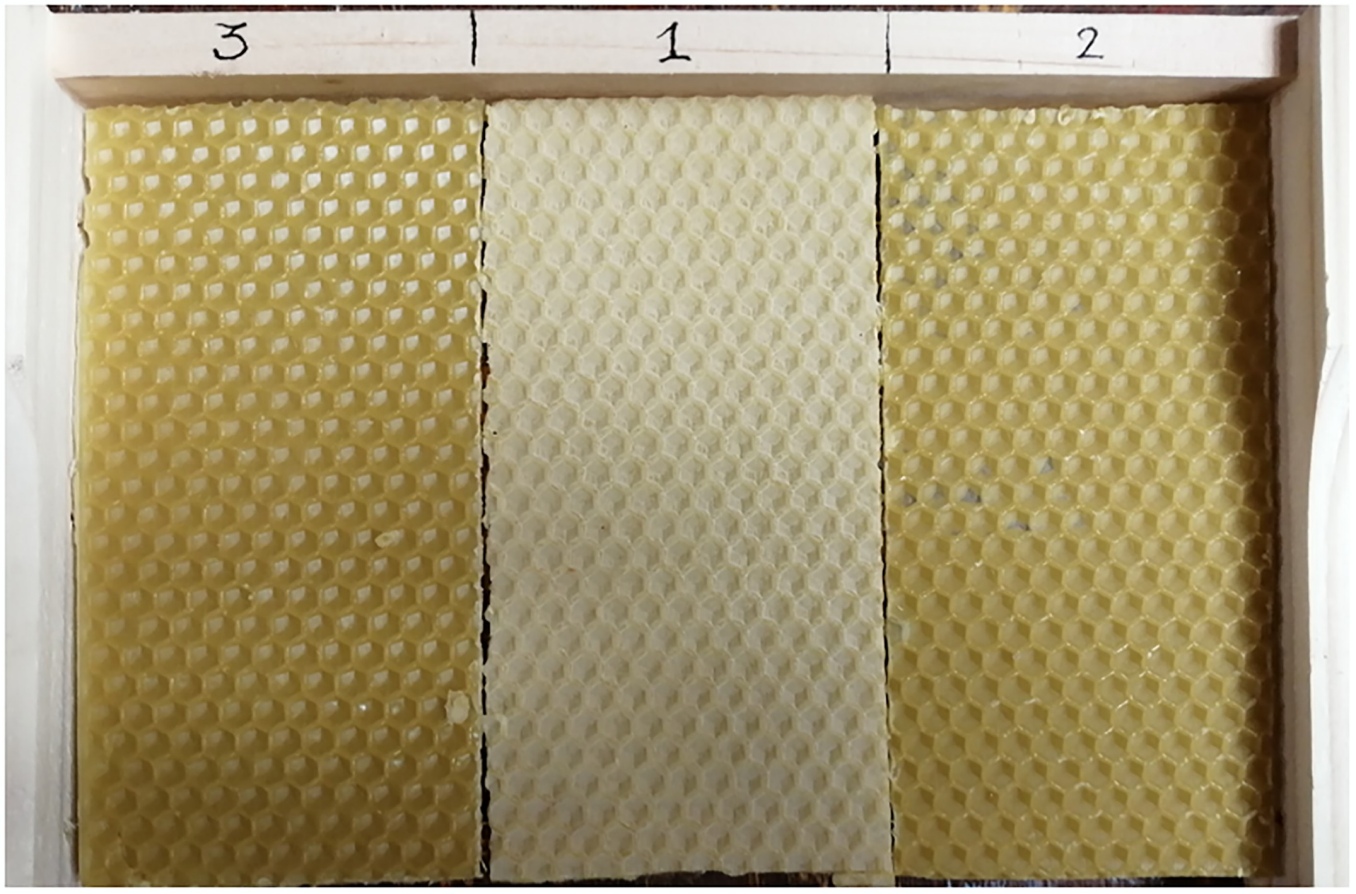
Mini Plus frame with a set of 3 comb sections, 2 contaminated and 1 control placed here in the centre.

Control and contaminated comb sections were placed randomly, side by side within the same frame (Fig 2). Per pesticide concentration or stearin percentage, six repetitions were carried out and each concentration was placed in one of the three positions in the frame. Each year, the frames were introduced at the same time in the hives, at the same period of the year and inspected daily to observe the date of the start of oviposition. The frames were then kept in the hive for 19 days after the first observation of oviposition to extract the pupae. Per substance concentration, three honey bee pupae were extracted randomly from each beeswax section, and stored at -80°C until analysis for gene expression.

### Reducing bias

To obtain same-age honey bee pupae in similar conditions and minimize the influence of external stressors such as pathogens and nutritional stress on gene expression, each year, healthy colonies of equal strength were newly formed on virgin beeswax foundations (to avoid cross-contaminations) with sister queens. Sister queens are the progeny of the same queen, which are mated at the same place to minimise genetic variability [50]. The colonies were cleared of the phoretic *Varroa* mite before their introduction in the Mini Plus hives. Per year and concentration, six repetitions were performed at the same time of the year. Experiments were conducted at the best time of the year for food sources abundance to avoid nutritional stress. The larvae stayed in the contaminated or adulterated beeswax for their entire development and were randomly sampled from the comb on day 19.

### Larval mortality rate estimation

**Fig 3:**
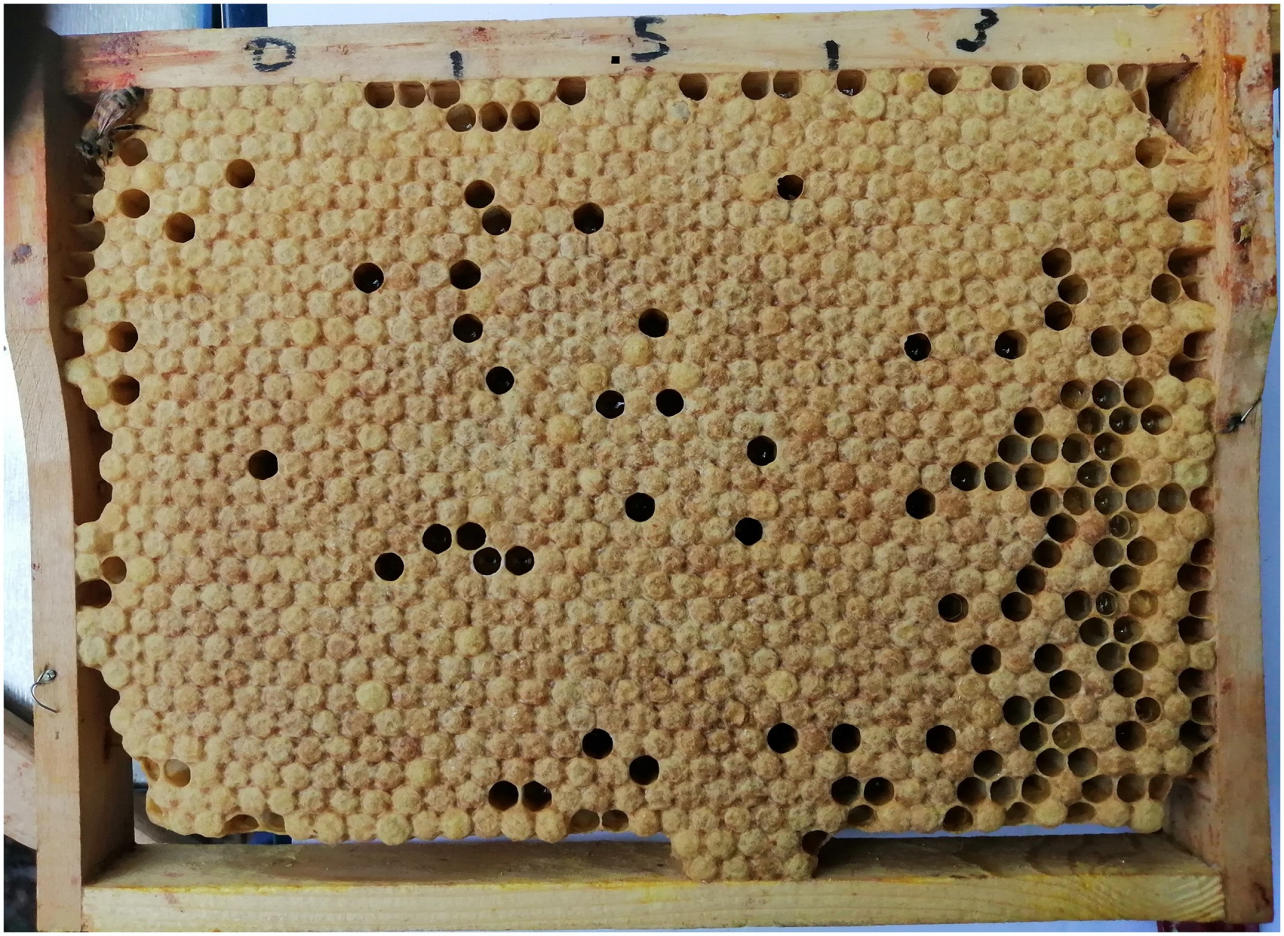
Brood frame with 3 percentages (0, 5 and 3%) of stearin showing empty cells for the accounting of larvae mortality rates. From left to right, the three percentages are 0, 5 and 3.

Within 4 days of frame introduction in the hive, and upon the start of oviposition, a piece of clear acetate was overlaid on the brood patch, and empty cells were marked. The total number of cells that contained larvae was counted, the acetate sheet was removed, and the frame was put back in the hive (Fig 3). On day 11, the prepupal survival rate was assessed by replacing the acetate sheet over the section of brood that was profiled on day 4 and noting any cells that had previously contained a larva but were then empty. Final mortality data were regressed on initial survival proportion [51].

### Gene expression

For the gene expression profiling, three frames from each treatment were selected and RNA was extracted from three individual pupae (randomly selected). The RNA extraction method as well as the quantification of gene expression of key proteins has been developed in a previous study by De Smet et al., 2017 [52]. Briefly, mechanical agitation using a TissueLyser (Precellys) for 90 s at 30 Hz, in the presence of a pair of stainless-steel beads and 1 ml Qiazol lysis reagent was used to homogenize the tissues of individual bees. The total RNA was extracted using RNeasy lipid tissue mini kit (Qiagen) and eluted in a final volume of 50μl. Total RNA concentration was measured using a Nanodrop (Isogen) equipment. The RevertAid H Minus First Strand cDNA Synthesis Kit was used to retrotranscribe 1 μg of total RNA using random hexamer primers (Thermo Scientific) Platinum (R) SYBR (R) Green qPCR Supermix-UDG (Live Technologies) was used for the RT-qPCR assays. Each 15 μl reaction consisted of 7.5 μl master mix, 0.2 μM forward and 0.2 μM reverse primer (Integrated DNA Technologies) and 0.2 μl cDNA template using the CFX96 Real-Time PCR Detection System (Bio-Rad). The PCR program included an activation step of 1 min at 95°C and 40 cycles of a combined denaturation (15 s at 95°C) and annealing step (30 s at 60°C). All reactions were performed in duplicate. No-template controls, containing diethylpyrocarbonate-treated water, were included in each run.

Using the geNormPLUS algorithm, the stability of the reference genes was analysed in the qBasePLUS environment (Biogazelle NV) with the default settings. A measure of stability (the M value) for each gene was generated by the geNorm program, enabling them to be ranked according to the stability of their expression (the lower the value, the greater the stability of the gene in the samples). To evaluate the benefit of integrating more reference genes in the normalization, a pairwise stability measure is generated. qPCR was used to ascertain the differential gene expression of 6 distinct target genes for immunity and detoxification. The primers used for these different genes are given in S1 Table. Relative expression levels were calculated by the comparative Delta-Ct method and were normalized to two reference genes (actin and RPL8) using the Biogazelle qBase+ software. Statistical analyses were done using qBase+ Statistics Wizard. The differential expression was analysed per exposure in which the different treatments were compared with the corresponding control group. The statistical analysis was performed using qBasePLUS, through one-way ANOVA. The correction for multiple testing and two-sided significance were carried out.

## Results

### Brood mortality rates

For all three tested substances, the larvae mortality rate was estimated by counting the number of uncapped cells after capping (after 11 days) with the exclusion of the unlaid cells. For Chlorpyriphos-ethyl, the mean brood mortality rate in the control wax for year one and year two are respectively 8.4 ± 1.83% and 10.3 ± 1.8% (see Table 1). The increase in concentration doesn’t specifically relate to a mortality rate increase.

**Table 1:** Mean mortality rates for chlorpyriphos-ethyl and mortality compared to control for the different concentrations tested.

Chlopyriphos-ethyl	Concentration in ppb	Mean mortality rate	SD	Mortality/Control
Year 1 (2020)	0	8.4%	1.83%	0%
	5	11.5%	2.52%	3.2%
	10	15.3%	1.9%	6.9%
Year 2 (2021)	0	10.3%	1.8%	0%
	500	14%	4.9%	3.7%
	5000	18.7%	4.3%	8.3%

For acrinathrin, the brood mortality rate in the control wax was 19.4 ± 8.3% for year one and 11.4 ± 4.7% for year two. Again, the increase in concentration doesn’t relate to a brood mortality rate increase (see Table 2).

**Table 2:** Mean mortality rates for acrinathrin and mortality compared to control for the different concentrations tested.

Acrinathrin	Concentration in ppb	Mean mortality rate	SD	Mortality/Control
Year 1 (2020)	0	19.4%	8.3%	0%
	12.5	24%	8.2%	4.54%
	25	24.5%	9.4%	5.14%
Year 2 (2021)	0	11.4%	4.7%	0%
	10	14.2%	3.6%	2.8%
	100	16.8%	8.1%	5.4%

**Fig 4:**
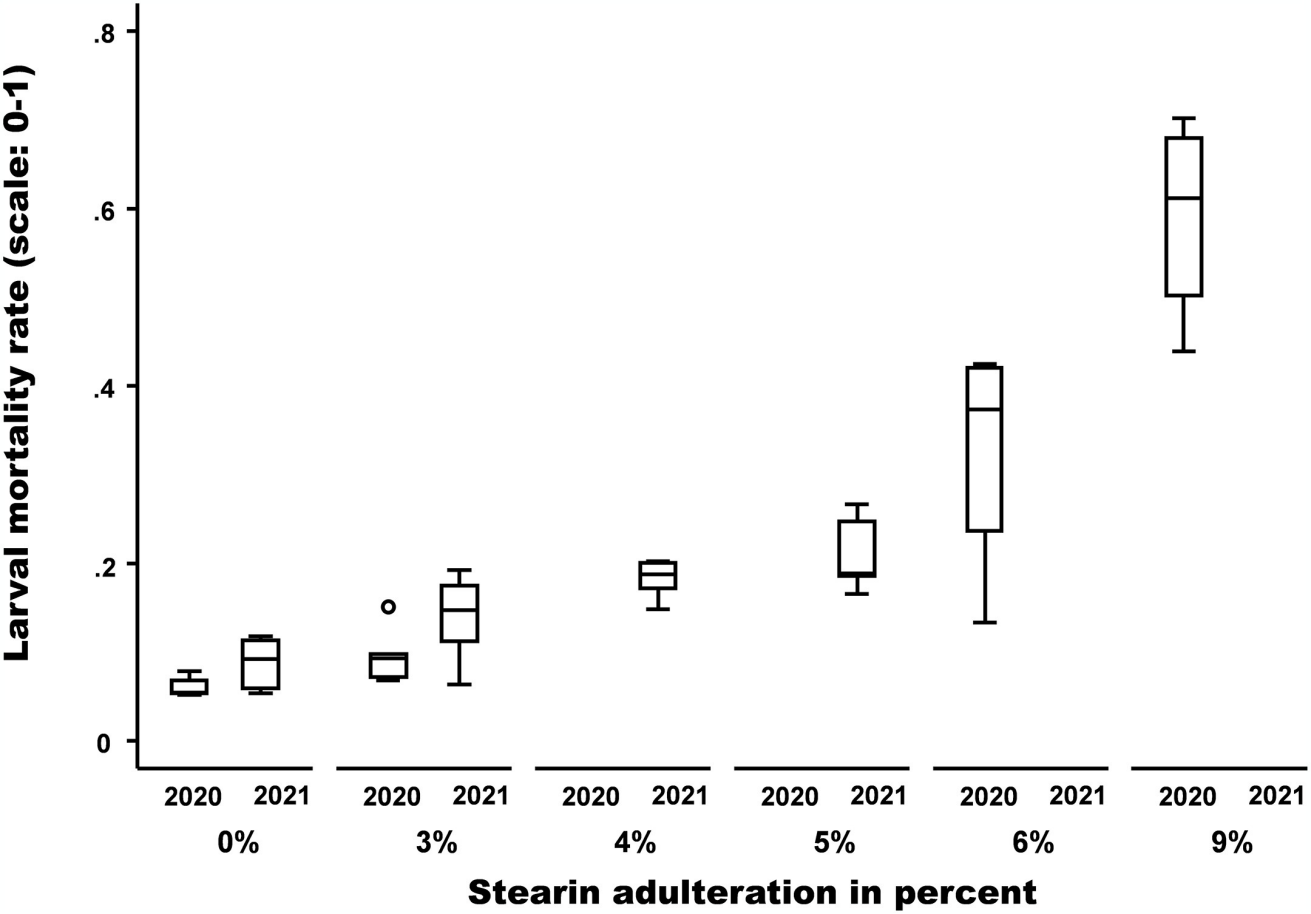
Increase of brood mortality with the increase of percentages of stearin in beeswax. Exponential brood mortality increase in function of the percentage of stearin adulteration (y = 0.0646e^*0*.*2389x*^; R^*2*^ = 0.7966).

With Stearin, the lowest mean brood mortality rates in the control frames were observed with a value of 6 ±1.06% for year one and 8.8 ±2.7% for year two (see Table 3). Unlike the tests with pesticides, brood mortality increased gradually with the increase of the stearin percentages in the beeswax. Fig 4 shows an exponential increase in brood mortality together with the increase in stearin percentages. From the addition of 3% of stearin, the brood mortality rate is no longer considered an acceptable level (<10%) [37].

**Table 3:** Mean mortality rates for Stearin and mortality compared to control for the different tested percentages.

Stearin	% Adulteration	Mean mortality rate	SD	Mortality/Control
Year 1 (2020)	0%	6%	1.06%	0%
	3%	13.2%	9.9%	3.6%
	6%	32.3%	13.1%	26.7%
	9%	59.1%	11.7%	53.1%
Year 2 (2021)	0%	8.8%	2.7%	0%
	3%	14%	4.7%	5.2%
	4%	18.2%	2.1%	9.4%
	5%	20.8%	4%	11.9%

To test if there are any statistical differences between the mean values of brood mortality rates for year one and year two, the non-parametric Kruskal-Wallis equality-of-populations rank test was used, comparing the brood mortality rates of the different concentrations throughout the years. Statistical differences in average mortality rate appeared in function of the concentration for chlorpyriphos-ethyl (χ2 = 28.24, d.f. = 4, P<0.0001), for acrinathrin (χ2 = 12.03, d.f. = 4, P<0.017) or stearin (χ2 = 35.5, d.f. = 6, P<0.0001).

### Genes expression effects quantification

**Fig 5:**
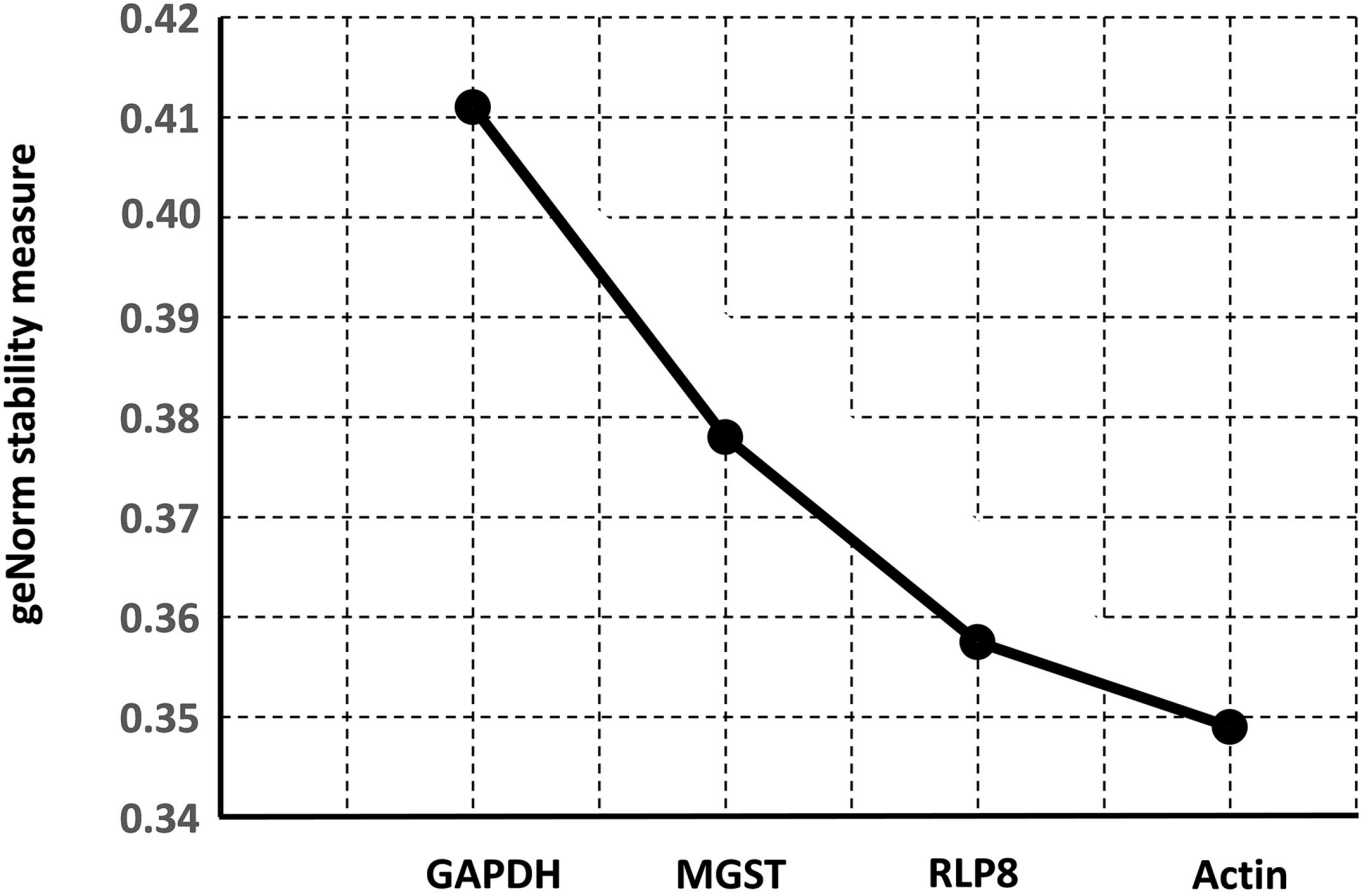
Average expression stability of reference targets with geNormPLUS algorithm.

The best and most reliable reference genes were chosen using the geNorm algorithm, and the four potential reference genes were ranked based on how stable they were for accurate gene expression. According to the data gathered from the various treatments, actin is the gene that is the most stable, followed by RPL8, MGST, and GADPH (Fig 5). Additionally, a pairwise stability measure is created to assess the value of including additional reference genes in the normalization. In this experimental design, two reference targets are the ideal number (geNorm V 0.15 compared to a normalization factor based on the two or three most stable targets). Thus, the geometric mean of the reference targets RLP8 and actin can be used to compute the ideal normalization factor.

### Exposure to acrinathrin

**Fig 6:**
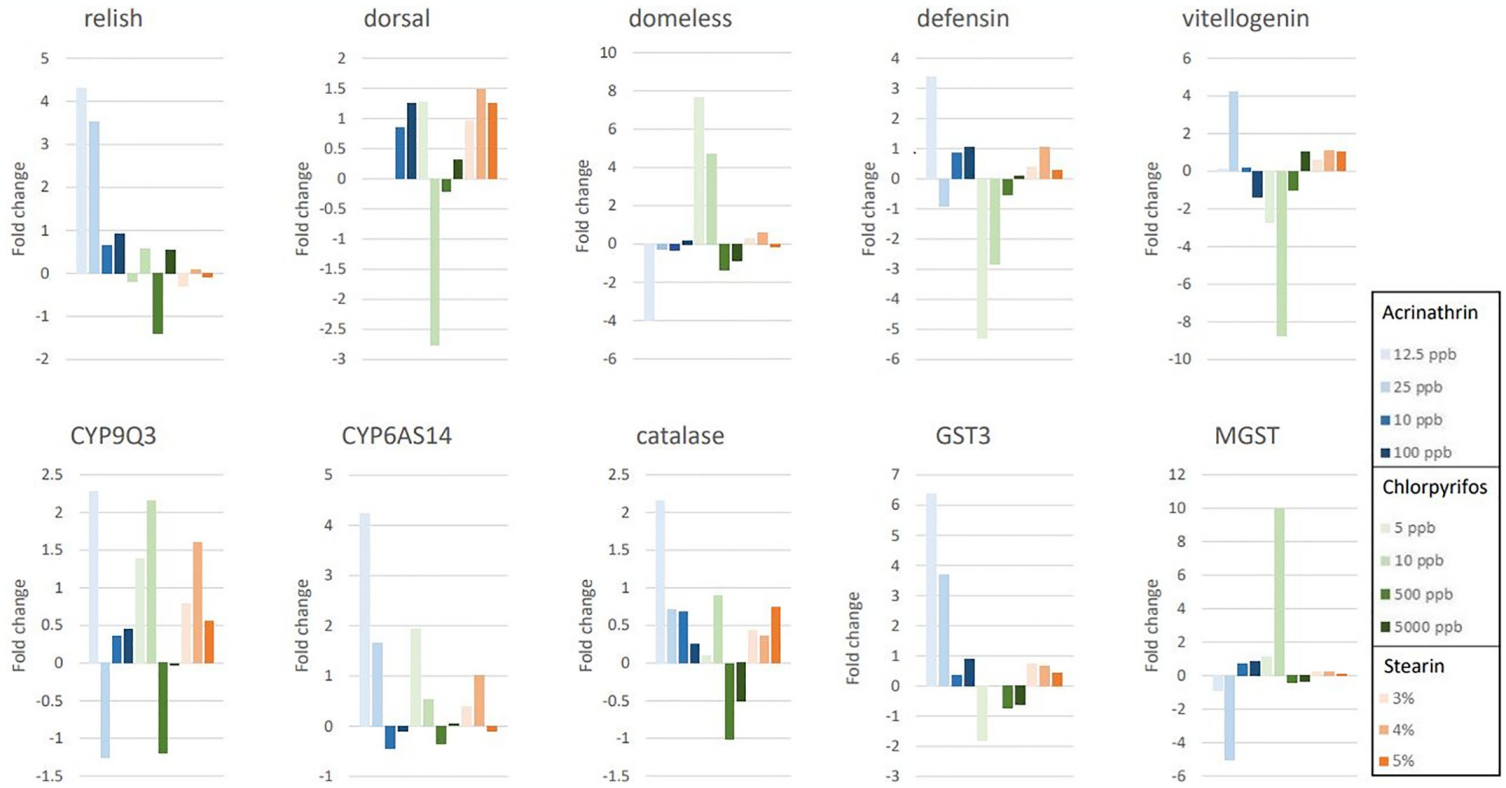
Expression profile of different immunity-related and detoxification genes in bee pupae after exposure to different concentrations of acrinathrin, chlorpyrifos-ethyl and stearin in beeswax (ppb or %).

Pupae raised in beeswax contaminated with 12.5 ppb and 25 ppb acrinathrin showed a significant upregulation of the *relish* gene when compared to the control group. The detoxification gene, CYP6AS14 was also significantly upregulated when pupae were exposed to beeswax containing 12.5 mg/kg acrinathrin. Exposure to 25 ppb showed an upregulation although not significant. Different immunity (*relish*, *defensin* and *dorsal*) and detoxification genes (CYP9Q3 and GST) were upregulated in pupae raised in beeswax contaminated with 10 and 100 ppb acrinathrin. The upregulation when compared to the control group was not significant. This shows that the immunity system of the bees was triggered by this contamination and that some detoxification processes were stimulated. The results are shown in Fig 6.

### Exposure to chlorpyrifos-ethyl

*Defensin 1* was downregulated when bee pupae were exposed to 5 and 10 ppb chlorpyrifos-ethyl in beeswax. However, the down-regulation was only significantly different from the control group when exposed to 5 ppb in beeswax. It is also worthwhile to notice that both detoxification genes, CYP6AS14 and CYP9Q3, were upregulated when the pupae were exposed to chlorpyrifos-ethyl. These upregulations were not significantly different from the control. When pupae were exposed to higher concentrations, 500 and 5000 ppb chlorpyrifos-ethyl in beeswax, most of the tested immunity genes and detoxification genes were downregulated. In pupae exposed to beeswax containing 500 ppb chlorpyrifos-ethyl, CYP9, GST3, *catalase* and *domeless* were significantly downregulated when compared with the control group. The results are shown in Fig 6. The downregulation of the immunity system is in line with the results when exposed to lower concentrations, although at these lower concentrations, the detoxification system was still active. The immunity system was also triggered at lower concentrations while at higher concentrations the bees seem to have suppressed immunity.

### Exposure to stearin

In the adulteration stearin experiment (3, 6 and 9%), no significant differences in gene expression were observed, compared to non-exposed pupae. Vitellogenin showed a very high level of expression when pupae were exposed to beeswax containing 3% stearin, but the variation in gene expression between the samples was also very high. Several genes were expressed at very low levels while the expression levels of the reference genes were normal in this first experiment. In a second experiment, the pupae were raised in beeswax contaminated with 3, 4 and 5% stearin. In contrast with the previous experiment, the expression levels of all tested genes were similar to the control. Almost all immunity genes and detoxification genes were upregulated which shows that the immunity system and detoxification processes were triggered by the exposure to stearin. CYP9Q3 and *dorsal* were significantly upregulated when exposed to 4% stearin in the beeswax during the pupation (Fig 6).

## Discussion

Pesticide residues as well as adulterants have multiple impacts on honey bee health, whether present in beeswax at acute or sublethal concentrations. Little is known about their impact on larval development and epigenetics. To verify if pollutants in beeswax have an impact on honey bee epigenetics we needed to reproduce, as accurately as possible, field conditions for wax contaminations as well as for honey bee rearing. Our study confirmed that honey bee immune response may either decrease or increase when exposed topically to pesticides or adulterants, posing a threat to honey bee health.

### Experimental setting

Contaminants are ubiquitous in beeswax and highly sensitive honey bee brood is exposed to it during its development. As shown by the recent study by Murcia Morales et al. 2020, residue transfer to bee brood occurs through direct contact with the beeswax [42]. Contaminating the wax mass aimed at reproducing realistic conditions, as beekeepers generally provide the hives with contaminated wax foundations. The wax foundations are used by the bees to build new comb, thus most of these contaminants are in the bottom of the bee cell, less in the cell walls, as they are thinner. As in the hive, contaminants are spread in the comb randomly.

Smaller colonies (Mini Plus hives) were used to conduct the field tests; Mini Plus hives are easier and quicker to handle, measure and observe. The system can sustain stable colonies that function as efficiently as larger colonies. Moreover, the assembly of study colonies is done to a high degree of standardisation in terms of brood quantity and adult bee population. For data verifiability and reproducibility, the basic units of the system, i.e., hive parts and frames, are available from beekeeping equipment suppliers [49]. Multiple colonies were used for the tests, this is recommended in risk assessments to better account for differences in stress responses at the colony level [53–56].

Field conditions test allowed the brood to develop in its natural environment, in the hive, without being disturbed by artificial test conditions, taking into account the effects on brood care by worker bees brood and the benefit from the colony effect (stress reduction). It seems that bees in field conditions can set up an immune reaction while bees housed in artificial cages suppress this reaction [52]. Placing control and treatment waxes within the same colony or frame during larval development evened out the effects of colony activity and the quality of resources fed to the brood.

Mean brood mortality rate in the control wax (see Table 1) should give us an indication of the quality of the implemented tests when compared to brood natural mortality rates. The scientific literature doesn’t say much about the natural brood mortality rates, but the study by Mortensen and Ellis, 2018 indicates brood mortality rates in field conditions at day 11, ≥ 20% [57] and the performance of the colonies in the control group in the study of Thompson et al., (2014) showed overall brood mortality rates between 10% and 30%, this demonstrates that the control group had performed correctly [58].

However, we cannot exclude potential environmental contaminations in beebread, thus the oral contaminations could have impacted the gene expression results. Nevertheless, the analytical results obtained were comparable, whatever repetition of the experiment, indicating a predominant effect of the tested pesticide on gene expression.

### Pesticide residues brood mortality rate

Although not much is known about the effects of chlorpyrifos-ethyl on honey bee brood, pupae appeared to be the most sensitive to chlorpyrifos (oral toxicity) out of five tested substances [40]. Our study showed higher brood mortality rates than the control colonies, with a non-linear increase in mortality vs. an increase in concentration. The concentrations used for the tests are far below the median acute lethal doses, and should rather have sublethal effects on honey bees. Chlorpyrifos is not used in apiculture, but this highly toxic organophosphate is one of the most ubiquitous chemicals found in hive matrices like beeswax.

Acrinathrin was used in apiculture to control the mite *Varroa jacobsoni*. It is still found at high concentrations in beeswax samples despite its high toxicity and developed resistance. Its transfer into larvae after direct contact with contaminated beeswax has been shown previously [42]. For acrinathrin, the concentrations used for the test were also below the acute contact lethal doses for honey bees, we observed higher mortality rates with the lowest concentrations (year one); the standard deviations of the mean mortality rates showed a greater disparity in the colonies tested the first year.

### Stearin brood mortality rates

With Stearin, brood mortality increased gradually with the increase of the stearin percentages in the beeswax. Fig 4 shows an exponential increase in brood mortality together with the increase in stearin percentages. These results are in line with the study of Chęć et al., 2021 [32]. In stearin-adulterated combs (10, 30 and 50% stearin), brood survival dropped significantly (P ≤ 0.001) compared with survival rates obtained in unadulterated wax foundations. The brood was scattered in the frame, probably due to the death of the young larvae, as a probable result of changes in the properties of the royal jelly [32]. From the addition of 3% of stearin, the brood mortality rate is no longer considered acceptable (<10%) [59].

### Gene expression analysis

The gene expression profiling of four genes involved in the major immune response to pathogens and eventually to environmental stress factors was performed: *relish* is involved in the Imd pathway; *domeless*, in the JAK-STAT pathway; and *dorsal*, in the Toll pathway and *defensin* can be used as a marker for antimicrobial peptide production [45, 60]. Next to the immune-related genes, some detoxification genes, CYP6AS14, and CYP9Q3 were also included. The detoxification process of the exposed pesticides and metabolites may be the initial process in neutralizing the chemicals. Exposure to pesticides may also lead to oxidative stress which can induce pathways involving catalase and GST to neutralize reactive oxygen species (ROS).

Our results suggest that exposure to acrinathrin is activating the immune system. At lower concentrations in beeswax the *relish* gene was significantly upregulated while at higher concentrations, *defensin* and *dorsal* were also upregulated although not significant. This likely reflects that acrinathrin is activating the Imd pathway leading to NF-κB activation. *Relish* regulates the expression of several antimicrobial peptide genes, such as *defensin* synthesis. The elevated *dorsal* expression suggests that next to the Imd pathway, the Toll pathway is also triggered in pupae raised in acrinathrin-contaminated beeswax at higher concentrations. This may also lead to the production of AMP-like *defensin* [61]. These results clearly show that the immune system of pupae raised in acrinathrin-contaminated beeswax is triggered, with possible negative impacts on colony health by making them more vulnerable to other stressors. However, further work is required to confirm this finding. Next to the triggered immune system, detoxification mechanisms were triggered to metabolize acrinathrin and the generated reactive oxygen species (ROS). Considering these results together, it seems that pupae react to acrinathrin exposure. The results also suggest that the bees try to metabolize acrinathrin using a CYP-dependent pathway.

Exposure to chlorpyrifos at low concentrations results in lowering the expression levels of *defensin*. At higher concentrations, the expression of *domeless* was significantly down-regulated. *Domeless* is a key enzyme in the JAK-STAT pathway, while *the* Imd and Toll pathways may regulate defensin expression. This expression profile suggests that the innate immune system is suppressed in pupae raised in chlorpyrifos-contaminated beeswax which will certainly lead to less resilient bees. Almost all expression levels of the tested immune and detoxification genes were down-regulated although not significantly different from the control. The expression profile suggests that bees are not or less able to neutralize chlorpyrifos and may be more vulnerable to pathogens and environmental stressors. This reaction can be explained by the lipophilic nature of chlorpyrifos and the lipid composition of the bee’s cuticle [62]. Lipophilic substances have a higher affinity for the cuticle, which makes them easier to absorb and more quickly delivered to their intended location of action [63]. This hypothesis formulated by Dorneles et al. evaluated organophosphorus pesticides toxicity to stingless bees and was based on the low water solubility of chlorpyrifos (1.05 mg/L at 20°C) [64]. More lipophilic compounds (i.e. less soluble in water) can penetrate more readily through the cuticle.

Exposure to stearin triggered the immune system and detoxification system of the pupae. CYP9Q3 and *dorsal* were significantly upregulated in the exposure experiment with 4% stearin. This likely reflects that the Toll pathway is activated and that the CYP-dependent detoxification mechanisms were initiated. As all immune-related genes were upregulated, although not significantly, this suggests that the pupae reacted to the presence of stearin which may harm their further health status, which should be studied in detail in future experiments.

All results of this study allow us to recommend gene expression as a tool to assess the sub-lethal effects of contaminated or adulterated beeswax on exposed worker pupae, using a novel *in vivo* realistic model.

## Conclusions

The ability to perform analysis of gene expression quantitatively has become increasingly important for animal health. To the best of our knowledge, this is the first field research that investigates the genomic responses of honey bees reared in beeswax contaminated with chlorpyrifos-ethyl, acrinathrin and adulterant stearin. Pesticide contaminations have an impact on honey bee mortality even at low concentrations, stearin adulteration of beeswax renders it inappropriate for use in beekeeping, as an addition of 3% of stearin significantly increases brood mortality. Our findings support the hypothesis that, at the tested concentrations of pesticide residues, honey bee immune response may either decrease or increase, posing a threat to honey bee health. As suggested by Dai et al. (2017), organophosphates (chlorpyrifos) seem to represent a higher risk to honey bee health than pyrethroids [40]. Further studies should be carried out to check whether adulterants have underlying effects. Further research on gene expression is crucial to understand the undelaying mode of actions of pesticides. For economically important and emblematic species such as honey bees, the identification of substance-specific response factors might ultimately serve to identify molecules that are safer for honey bees and the ecosystem’s health.

## Supporting information

S1 Table Characteristics of the RT-qPCR analysis of differentially expressed genes of *Apis mellifera*. (DOCX)

## References

[1] Al-Kahtani SN, Taha EK A. Effect of comb age on cell measurements and worker body size. PLoS One. 2021, 16, 11–13. doi: 10.1371/journal.pone.0260865 34860846 PMC8641860

[2] Wilmart O, Legrève A, Scippo M-L, Reybroeck W, Urbain B, de Graaf DC, et al. Honey bee exposure scenarios to selected residues through contaminated beeswax. Sci Total Environ. 2021, 772, 145533. doi: 10.1016/j.scitotenv.2021.145533 33770874

[3] Calatayud-Vernich P, Calatayud F, Simó E, Picó Y. Pesticide residues in honey bees, pollen and beeswax: Assessing beehive exposure. Environ Pollut. 2018, 241, 106–114. doi: 10.1016/j.envpol.2018.05.062 29803024

[4] Daniele G, Giroud B, Jabot C, Vulliet E. Exposure assessment of honeybees through study of hive matrices: analysis of selected pesticide residues in honeybees, beebread, and beeswax from French beehives by LC-MS/MS. Environ Sci Pollut Res Int. 2018, 25(7), 6145–6153. doi: 10.1007/s11356-017-9227-7 28560623

[5] Mullin CA, Frazier M, Frazier JL, Ashcraft S, Simonds R, Vanengelsdorp D, et al. High levels of miticides and agrochemicals in North American apiaries: implications for honey bee health. PLoS One. 2010, 5(3), e9754. doi: 10.1371/journal.pone.0009754 20333298 PMC2841636

[6] Piechowicz B, Mróz K, Szpyrka E, Zwolak A, Grodzicki P. Transfer of plant protection products from raspberry crops of Laszka and Seedling varieties to beehives. Environ Monit Assess. 2018, 190(3), 135. doi: 10.1007/s10661-018-6491-z 29435675 PMC5809555

[7] Tong Z, Duan J, Wu Y, Liu Q, He Q, Shi Y, et al. A survey of multiple pesticide residues in pollen and beebread collected in China. Sci Total Environ. 2018, 640–641, 1578–1586. doi: 10.1016/j.scitotenv.2018.04.424 30021322

[8] Traynor KS, Pettis JS, Tarpy DR, Mullin CA, Frazier JL, Frazier M, et al. In-hive Pesticide Exposome: Assessing risks to migratory honey bees from in-hive pesticide contamination in the Eastern United States. Sci Rep. 2016, 6(1), 33207. doi: 10.1038/srep33207 27628343 PMC5024099

[9] Martel A-C, Zeggane S, Eres C, Drajnudel P, Faucon J, Aubert M, et al. Acaricide residues in honey and wax after treatment of honey bee colonies with Apivar or Asuntol 50. Apidologie. 2007, 38, 53444. https://hal.archives-ouvertes.fr/hal-00892290.

[10] Sanchez-Bayo F, Goka K. Pesticide residues and bees—A risk assessment. PLoS One. 2014, 9(4), e94482. doi: 10.1371/journal.pone.0094482 24718419 PMC3981812

[11] Perugini M, Tulini SMR, Zezza D, Fenucci S, Conte A, Amorena M. Occurrence of agrochemical residues in beeswax samples collected in Italy during 2013–2015. Sci Total Environ. 2018, 625:470–476. doi: 10.1016/j.scitotenv.2017.12.321/. 29291561

[12] Suchail S, Guez D, Belzunces LP. Discrepancy Between Acute and Chronic Toxicity Induced By Imidacloprid and Its Metabolites in Apis Mellifera. Environ Toxicol Chem.,. 2001, 20(11), 2482–2486. doi: 10.1897/1551-5028(2001)020&lt;2482:dbaact&gt;2.0.co;2 11699773

[13] Weick J, Thorn RS. Effects of Acute Sublethal Exposure to Coumaphos or Diazinon on Acquisition and Discrimination of Odor Stimuli in the Honey Bee (Hymenoptera: Apidae). J Econ Entomol. 2002, 95(2), 227–236. doi: 10.1603/0022-0493-95.2.227 12019994

[14] Yang W, Chang J, Xu B, Peng C, Ge Y. Ecosystem service value assessment for constructed wetlands: A case study in Hangzhou, China. Ecol Econ. 2008, 8. doi: 10.1016/j.ecolecon.2008.02.008

[15] Aliouane Y, el Hassani AK, Gary V, Armengaud C, Lambin M, Gauthier M. Subchronic Exposure of Honeybees To Sublethal Doses of Pesticides: Effects on Behavior. Environ Toxicol Chem. 2009, 28(1), 113. doi: 10.1897/08-110.1 18700810

[16] Desneux N, Decourtye A, Delpuech J-M. The Sublethal Effects of Pesticides on Beneficial Arthropods. Annu Rev Entomol. 2007, 52(1), 81–106. doi: 10.1146/annurev.ento.52.110405.091440 16842032

[17] Bogdanov S. Beeswax: Uses and Trade. In: The Beeswax Book. 2009. p. 1–16.

[18] Bogdanov S. Beeswax: Production, Properties, Composition, Control. In: Beeswax book. Muehlethurnen, Switzerland: Bee Product Science Publishing; 2016. p. 1–17. www.bee-hexagon.net

[19] Waś E, Szczęsna T, Rybak-Chmielewska H. Efficiency of GC-MS method in detection of beeswax adulterated with paraffin. J Apic Sci. 2016, 60(1), 145–161. doi: doi.org/10.1515/jas-2016-0012

[20] Svečnjak L, Jović O, Prđun S, Rogina J, Marijanović Z, Car J, et al. Influence of beeswax adulteration with paraffin on the composition and quality of honey determined by physico-chemical analyses, 1H NMR, FTIR-ATR and HS-SPME/GC–MS. Food Chem. 2019, 291, 187–198. https://www.sciencedirect.com/science/article/pii/S0308814619306466?via%3Dihub 31006458 10.1016/j.foodchem.2019.03.151

[21] Tulloch AP. Factors affecting analytical values of beeswax and detection of adulteration. J Am Oil Chem Soc. 1973, 50(7), 269–72. doi: 10.1007/BF02641800

[22] Bogdanov S. Beeswax: Quality issues today. Bee World. 2004, 85(3), 46–50. doi: 10.1080/0005772X.2004.11099623

[23] Svečnjak L, Baranović G, Vinceković M, Prđun S, Bubalo D, Gajger IT. An approach for routine analytical detection of beeswax adulteration using FTIR-ATR spectroscopy. J Apic Sci. 2015, 59(2), 37–49. doi: 10.1515/jas-2015-0018

[24] Špaldoňová A, Havelcová M, Lapčák L, Machovič V, Titěra D. Analysis of beeswax adulteration with paraffin using GC/MS, FTIR-ATR and Raman spectroscopy. J Apic Res. 2021, 60(1), 73–83. doi: 10.1080/00218839.2020.1774152

[25] El Agrebi N, Svečnjak L, Horvatinec J, Renault V, Rortais A, Cravedi J-P, et al. Adulteration of beeswax: A first nationwide survey from Belgium. PLoS One. 2021, 16(9), e0252806. doi: 10.1371/journal.pone.0252806 34499645 PMC8428765

[26] El Agrebi N, Traynor K, Wilmart O, Tosi S, Leinartz L, Danneels E, et al. Pesticide and veterinary drug residues in Belgian beeswax: Occurrence, toxicity, and risk to honey bees. Sci Total Environ. 2020, 745, 141036. doi: 10.1016/j.scitotenv.2020.141036 32758732

[27] Wilmart O, Legrève A, Scippo M-L, Reybroeck W, Urbain B, de Graaf DC, et al. Residues in Beeswax: A Health Risk for the Consumer of Honey and Beeswax? J Agric Food Chem. 2016, 64(44), 8425–8434. doi: 10.1021/acs.jafc.6b02813 27741395

[28] Svečnjak L. Alarming situation on the EU beeswax market: the prevalence of adulterated beeswax material and related safety issues. In: Program and Abstracts Book EurBee 8th Congress of Audiology, Ghent, Belgium, 18–20 September 2018. 2018. p. 114–5.

[29] Bernal JL, Jiménez JJ, del Nozal MJ, Toribio L, Martín MT. Physico-chemical parameters for the characterization of pure beeswax and detection of adulterations. Eur J Lipid Sci Technol. 2005, 107(3), 158–66. doi: 10.1002/ejlt.200401105

[30] Tanner N, Lichtenberg-Kraag B. Identification and Quantification of Single and Multi-Adulteration of Beeswax by FTIR-ATR Spectroscopy. Eur J Lipid Sci Technol. 2019, 121(12), 1–10. doi: 10.1002/ejlt.201900245

[31] Maia M, Barros AIRNA, Nunes FM. A novel, direct, reagent-free method for the detection of beeswax adulteration by single-reflection attenuated total reflectance mid-infrared spectroscopy. Talanta, 2013, 107, 74–80. doi: 10.1016/j.talanta.2012.09.052 23598195

[32] Chęć MCH, Lszewski KO, Ziechciarz PD, Kowronek PS, Ietrow MP, Orsuk GB, et al. Effect of stearin and paraffin adulteration of beeswax on brood survival. Apidologie. 2021, 52, 432–446. doi: 10.1007/s13592-020-00833-7

[33] Reybroeck W. F trial: effect of the addition of stearic and palmitic acid to beeswax on the development of the worker bee brood [Internet]. 2018. https://www.health.belgium.be/sites/default/files/uploads/fields/fpshealth_theme_file/verslag_veldproef_ilvo_2018_eng.pd

[34] Payne AN, Walsh EM, Rangel J. Initial Exposure of Wax Foundation to Agrochemicals Causes Negligible Effects on the Growth and Winter Survival of Incipient Honey Bee (Apis mellifera) Colonies. Insects, 2019, 10(1), 19. doi: 10.3390/insects10010019 30626042 PMC6359559

[35] Al Naggar Y, Codling G, Vogt A, Naiem E, Mona M, Seif A, et al. Organophosphorus insecticides in honey, pollen and bees (Apis mellifera L.) and their potential hazard to bee colonies in Egypt. Ecotoxicol Environ Saf., 2015, 114, 1–8. doi: 10.1016/j.ecoenv.2014.12.039 25574845

[36] Tosi S, Costa C, Vesco U, Quaglia G, Guido G. A 3-year survey of Italian honey bee-collected pollen reveals widespread contamination by agricultural pesticides. Sci Total Environ., 2018, 615:208–218. doi: 10.1016/j.scitotenv.2017.09.226 28968582

[37] Casida JE, Durkin KA. Neuroactive insecticides: Targets, selectivity, resistance, and secondary effects. Annu Rev Entomol. 2013;58:99–117. doi: 10.1146/annurev-ento-120811-153645 23317040

[38] Gregorc A, Ellis JD. Cell death localization in situ in laboratory reared honey bee (Apis mellifera L.) larvae treated with pesticides. Pestic Biochem Physiol. 2011, 99(2), 200–207. doi: 10.1016/j.pestbp.2010.12.005

[39] Fisher A, Rangel J. Exposure to pesticides during development negatively affects honey bee (Apis mellifera) drone sperm viability. PLoS One. 2018, 13(12), e0208630. doi: 10.1371/journal.pone.0208630 30543709 PMC6292656

[40] Dai P, Jack CJ, Mortensen AN, Ellis JD. Acute toxicity of five pesticides to Apis mellifera larvae reared in vitro. Pest Manag Sci. 2017, 73(11), 2282–2286. doi: 10.1002/ps.4608 28485079

[41] Dai P, Jack CJ, Mortensen AN, Bustamante TA, Bloomquist JR, Ellis JD. Chronic toxicity of clothianidin, imidacloprid, chlorpyrifos, and dimethoate to Apis mellifera L. larvae reared in vitro. Pest Manag Sci. 2019, 75(1), 29–36. doi: 10.1002/ps.5124 29931787

[42] Murcia Morales M, Gómez Ramos MJ, Parrilla Vázquez P, Díaz Galiano FJ, García Valverde M, Gámiz López V, et al. Distribution of chemical residues in the beehive compartments and their transfer to the honeybee brood. Sci Total Environ. 2020, 710, 136288. doi: 10.1016/j.scitotenv.2019.136288 31927284

[43] EFSA. Conclusion on the peer review of the pesticide risk assessment of the active substance acrinathrin. EFSA J. 2013, 11(12), 3469. http://www.efsa.europa.eu/efsajournal 10.2903/j.efsa.2010.1872 PMC13085163 42004101

[44] El Agrebi N, Svečnjak L. Adulteration of Belgian beeswax: a first nationwide survey. PLoS One. 2021, 16(9), e0252806. doi: 10.1371/journal.pone.0252806 34499645 PMC8428765

[45] Brutscher LM, Daughenbaugh KF, Flenniken ML. Antiviral defense mechanisms in honey bees. Curr Opin Insect Sci., 2015, 10, 71–82. doi: 10.1016/j.cois.2015.04.016 26273564 PMC4530548

[46] Calatayud-Vernich P, Calatayud F, Simó E, Picó Y. Occurrence of pesticide residues in Spanish beeswax. Sci Total Environ. 2017, 605–606, 745–754. doi: 10.1016/j.scitotenv.2017.06.174 28679118

[47] Serra-Bonvehí J, Orantes-Bermejo J. Acaricides and their residues in Spanish commercial beeswax. Pest Manag Sci. 2010, 66(11), 1230–1235. doi: 10.1002/ps.1999 20661942

[48] Marti JNG, Kilchenmann V, Kast C. Evaluation of pesticide residues in commercial Swiss beeswax collected in 2019 using ultra—high performance liquid chromatographic analysis. Environ Sci Pollut Res. 2022, 0123456789. doi: 10.1007/s11356-021-18363-9 35018599 PMC9054900

[49] Allan MJ, Dean RR. An integrated system for field studies on honey bees. J Apic Res. 2022, 61(3), 317–319. doi: 10.1080/00218839.2021.2018107

[50] OECD. Guidance document on the honey bee (Apis Mellifera L.) brood test under semi-field conditions. 2007.

[51] Human H, Brodschneider R, Dietemann V, Dively G, Ellis JD, Forsgren E, et al. Miscellaneous standard methods for Apis mellifera research. J Apic Res. 2013, 52(4), doi: 10.3896/IBRA.1.52.4.10

[52] De Smet L., Hatjina F., Ioannidis P., Hamamtzoglou A., Schoonvaere K., Francis F., et al. Stress indicator gene expression profiles, colony dynamics and tissue development of honey bees exposed to sub-lethal doses of imidacloprid in laboratory and field experiments. PLoS One, 2017, 12, e0171529. doi: 10.1371/journal.pone.0171529 28182641 PMC5300173

[53] Alix A, Chauzat MP, Duchard S, Lewis G, Maus C, Miles MJ, et al. Chapter 10: Honeybees–Proposed scheme. In: Environmental risk assessment scheme for plant protection products. 2009.

[54] Crailsheim K, Brodschneider R, Aupinel P, Behrens D, Genersch E, Vollmann J, et al. Standard methods for artificial rearing of Apis mellifera larvae. J Apic Res. 2013, 52(1), 15. doi: 10.3896/IBRA.1.52.1.05

[55] OECD. Guidance Document on Honey Bee (Apis mellifera) Larval Toxicity Test, Repeated Exposure. 2016. https://one.oecd.org/document/ENV/JM/MONO(2016)34/en/pdf.

[56] De Smet L, Hatjina F, Ioannidis P, Hamamtzoglou A, Schoonvaere K, Francis F, et al. Stress indicator gene expression profiles, colony dynamics and tissue development of honey bees exposed to sub-lethal doses of imidacloprid in laboratory and field experiments. Riechers DE, editor. PLoS One. 2017, 12(2), e0171529. doi: 10.1371/journal.pone.0171529 28182641 PMC5300173

[57] Mortensen AN, Ellis JD. A honey bee (Apis mellifera) colony’s brood survival rate predicts its in vitro-reared brood survival rate. Apidologie. 2018, 49(5), 573–580. doi: 10.1007/s13592-018-0584-0

[58] Thompson HM, Levine SL, Doering J, Norman S, Manson P, Sutton P, et al. Evaluating exposure and potential effects on honeybee brood (Apis mellifera) development using glyphosate as an example. Integr Environ Assess Manag. 2014, 10(3), 463–470. doi: 10.1002/ieam.1529 24616275 PMC4285224

[59] El Agrebi N, Steinhauer N, Renault V, de Graaf DC, Saegerman C. Beekeepers perception of risks affecting colony loss: A pilot survey. Transbound Emerg Dis. 2022, 69(2), 579–590. doi: 10.1111/tbed.14023 33544964

[60] Barroso-Arévalo S, Vicente-Rubiano M, Puerta F, Molero F, Sánchez-Vizcaíno JM. Immune related genes as markers for monitoring health status of honey bee colonies. BMC Vet Res., 2019, 15(1), 72. doi: 10.1186/s12917-019-1823-y 30832657 PMC6398266

[61] Schlüns H, Crozier RH. Relish regulates expression of antimicrobial peptide genes in the honeybee, Apis mellifera, shown by RNA interference. Insect Mol Biol. 2007, 16(6), 753–759. doi: 10.1111/j.1365-2583.2007.00768.x 18093004

[62] Bacci L, Pereira EJG, Fernandes FL, Picanço MC, Crespo ALB, Mateus E, et al. Physiological Selectivity of Insecticides to Predator Wasps (Hymenoptera: Vespidae) of Leucoptera coffeella (Lepidoptera: Lyonetiidae). BioAssay. 2006, 1(10), 1–7. www.seb.org.br/bioassay

[63] Leite GLD, Picanço M, Guedes RNC, Gusmão MR. Selectivity of insecticides with and without mineral oil to Brachygastra lecheguana (Hymenoptera: Vespidae), a predator of Tuta absoluta (Lepidoptera: Gelechiidae). Ceiba. 1998;39(2):191–4.

[64] Dorneles AL, de Souza Rosa A, Blochtein B. Toxicity of organophosphorus pesticides to the stingless bees Scaptotrigona bipunctata and Tetragonisca fiebrigi. Apidologie. 2017, 48(5), 612–620. doi: 10.1007/s13592-017-0502-x

