# Supplementary material for: A field realistic model to assess the effects of pesticides residues and adulterants on honey bee gene expression
Source: PLoS One. 2024 Jun 26;19(6):e0302183. doi: 10.1371/journal.pone.0302183 (PMC11206931; doi:10.1371/journal.pone.0302183)
Supplement: S1 Table — (DOCX) [file pone.0302183.s001.docx]

**S1 Table.** Characteristics of the RT-qPCR analysis of differentially expressed genes of Apis mellifera

|  | Sequence (5’-3’) | References |
| --- | --- | --- |
| Reference genes | | |
| *Actin* | F: TGCCAACACTGTCCTTTCTG  R: AGAATTGACCCACCAATCCA | Cunha et al., 2005 |
| RLP8 | F: TGGATGTTCAACAGGGTTCATA  R: CTGGTGGTGGACGTATTGATAA | Evans, 2006 |
| MGST | F: TTGCTCTGTAAGGTTGTTTTGC  R: TGTCTGGTTAACTACAAATCCTCCTG | Cornman et al., 2012 |
| GAPDH | F: GATGCACCCATGTTTGTTTG  R:TTTGCAGAAGGTGCATCAAC | Scharlaken et al., 2008 |
| Target genes | | |
| GST3 | F: TGCATATGCTGGCATTGATT R: TCCTCGCCAAGTATCTTGCT | Gregorc et al., 2012 |
| CYP6AS14 | F: TGAAACTCATGACCGAGACG  R: AAAATTTGGGCCGCTAATAAA | Al Naggar et al., 2015 |
| CYP9Q3 | F: GTAGCCATTCACGCGTTCAC  R: GTCTCGTCGATCTCCTGCTG | De Smet et al., 2017 |
| *Catalase* | F: GGCGGCTGAATTAAGTGCTA  R: TTGCGTTGTGTTGGAGTCAT | Collins et al., 2004 |
| *Relish* | F: GCAGTGTTGAAGGAGCTGAA  R: CCAATTCTGAAAAGCGTCCA | Evans, 2006 |
| *Domeless* | F: TTGTGCTCCTGAAAATGCTG  R: AACCTCCAAATCGCTCTGTG | Evans, 2006 |
| *Dorsal-2* | F: TCACCATCAACGCCTAACAA  R: AACTAACACCACGCGCTTCT | Evans, 2006 |
| *Defensin-1* | F: TGCGCTGCTAACTGTCTCAG  R: AATGGCACTTAACCGAAACG | Evans, 2006 |
| *Vitellogenin* | F: ACGTAATAAATGCCGCCAAG  R: TGCATGTTGCTCTCCAACTC | De Smet et al., 2017 |

Al Naggar, Y., Codling, G., Vogt, A., Naiem, E., Mona, M., Seif, A., Giesy, J.P. Organophosphorus insecticides in honey, pollen and bees (Apis mellifera L.) and their potential hazard to bee colonies in Egypt. Ecotoxicol. Environ. Saf. 2015, 114, 1-8. [doi: 10.1016/j.ecoenv.2014.12.039](https://doi.org/10.1016/j.ecoenv.2014.12.039).

Collins, A.M., Williams, V., Evans, J.D. Sperm storage and antioxidative enzyme expression in the honey bee, Apis mellifera. Insect Mol. Biol., 2004, 13, 141-146. [doi: 10.1111/j.0962-1075.2004.00469.x](https://doi.org/10.1111/j.0962-1075.2004.00469.x)

Cornman, R.S., Tarpy, D.R., Chen, Y., Jeffreys, L., Lopez, D., Pettis, J.S., vanEngelsdorp, D., Evans, J.D. Pathogen webs in collapsing honey bee colonies. PLoS One 2012, 7, e43562. doi: 10.1371/journal.pone.0043562.

Cunha, A.D., Nascimento, A.M., Guidugli, K.R., Simões, Z.L.P., Bitondi, M.M.G., 2005. Molecular cloning and expression of a hexamerin cDNA from the honey bee, Apis mellifera. J. Insect Physiol. 51, 1135-1147. doi: 10.1016/j.jinsphys.2005.06.004.

De Smet, L., Hatjina, F., Ioannidis, P., Hamamtzoglou, A., Schoonvaere, K., Francis, F., Meeus, I., Smagghe, G., de Graaf, D.C. Stress indicator gene expression profiles, colony dynamics and tissue development of honey bees exposed to sub-lethal doses of imidacloprid in laboratory and field experiments. PLoS One, 2017, 12, e0171529. doi: 10.1371/journal.pone.0171529.

Evans, J.D., 2006. Beepath: An ordered quantitative-PCR array for exploring honey bee immunity and disease. J. Invertebr. Pathol. 93, 135–139. doi: 10.1016/j.jip.2006.04.004.

Gregorc, A., Ellis, J.D. Cell death localization in situ in laboratory reared honey bee (Apis mellifera L.) larvae treated with pesticides. Pestic. Biochem. Physiol., 2011 99, 200-207. doi: 10.1016/J.PESTBP.2010.12.005.

Scharlaken, B., De Graaf, D.C., Goossens, K., Brunain, M., Peelman, L.J., Jacobs, F.J., Reference gene selection for insect expression studies using quantitative real-time PCR: The head of the honeybee, Apis mellifera, after a bacterial challenge. J. Insect Sci., 2008, 8, 33. 33. doi: 10.1673/031.008.3301.
